# Genome-wide association mapping and accuracy of predictions for amoebic gill disease in Atlantic salmon (*Salmo salar*)

**DOI:** 10.1038/s41598-020-63423-8

**Published:** 2020-04-15

**Authors:** Muhammad L. Aslam, Solomon A. Boison, Marie Lillehammer, Ashie Norris, Bjarne Gjerde

**Affiliations:** 1 Department of Breeding and Genetics, Nofima AS, P.O. Box 210, N-1431 Ås, Norway; 2 Marine Harvest ASA (old name) with new name Mowi Genetics AS, 5035 Bergen, Norway

**Keywords:** Animal breeding, Genome-wide association studies, Quantitative trait loci

## Abstract

Amoebic gill disease (AGD) is a parasitic disease caused by the amoeba *Paramoeba perurans*, which colonizes the gill tissues and causes distress for the host. AGD can cause high morbidity and mortalities in salmonid and non-salmonid fish species. To understand the genetic basis of AGD and improve health status of farmed A. salmon, a population of ~ 6,100 individuals belonging to 150 full-sib families was monitored for development of AGD in the sea of Ireland. The population was followed for two rounds of AGD infections, and fish were gill scored to identify severity of disease in first (N = 3,663) and the second (N = 3,511) infection with freshwater treatment after the first gill-scoring. A subset of this gill-scored population (N = 1,141) from 119 full-sib families were genotyped with 57,184 SNPs using custom-made Affymetrix SNP-chip. GWAS analyses were performed which resulted in five significantly associated SNP variants distributed over chromosome 1, 2 and 5. Three candidate genes; *c4, tnxb* and *slc44a4* were found within QTL region of chromosome 2. The *tnxb* and *c4* genes are known to be a part of innate immune system, and may play a role in resistance to AGD. The gain in prediction accuracy obtained by involving genomic information was 9–17% higher than using traditional pedigree information.

## Introduction

Amoebic gill disease (AGD) is caused by a parasite *Paramoeba perurans*, which colonizes gill tissue^1,2^ and ultimately causes inappetence, respiratory distress and cardiovascular compromise^3,4^. Attachment of amoebae to the gill initiates a localized host cellular response, including hyperplasia and hypertrophy of the gill epithelium and lamellar fusion^5^. This pathological condition can cause high production losses in multiple salmonid and non-salmonid fish species^3,4^. Although AGD can occur year-round, it is most prevalent in warmer water and high salinity with increased frequency and severity^6^. The existence and severity of disease at farmed facilities is evaluated by random sampling of fish (~10–15) and investigation and/or scoring of gills for pathological conditions caused by amoeba as described by Taylor *et al*.^7^. The detection for the specific type of pathogen/amoeba and the pathogen load (measure of disease severity) can also be determined using quantitative PCR (qPCR) where load of pathogen is determined using threshold cycle (***C***_***t***_) values^8–10^. However, studies have shown that the severity of AGD established using Taylor scoring system vs. ***C***_***t***_ values using qPCR have high genetic (close to unity) and phenotypic (0.81) correlations revealing that both are potentially the same trait^8,10^.

Amoebic gill disease has been a major problem over many years in farmed Atlantic salmon (*Salmo salar*) of Tasmania, and cleaning of amoeba from the gills requires freshwater treatments which costs ~10–20% of the total production cost^4^. The treatment is also a welfare issue for the fish due to the physiological stress caused by freshwater bath.

In Northern Atlantic, the presence of *P. perurans* has been documented for more than a decade, but for a long time the cold water seemed to have prevented an epidemic of AGD^3^. However, warm and dry weather conditions in 2011 and 2012 for Ireland and Scotland, and later in 2012–2013, at Northern Isles (Orkney and Shetland), Norway and the Faroe Islands caused major AGD outbreaks on farmed Atlantic salmon^11^, and AGD became the largest infectious health problem for the salmon industry in Ireland, Scotland and France those years^12^.

AGD is a rising threat for Norwegian salmon with first documented occurrence in 2006^13^, and since then amoeba has been regularly reported every year on the southwest coast and further north^14^ in Norway.

Norwegian Atlantic salmon populations from the two breeding companies (Marine Harvest ASA and SalmoBreed AS) have shown genetic variation for resistance against AGD both in field (${h}^{2}$ of 0.12–0.20) and challenge test (${h}^{2}$ of 0.09–0.13) conditions^15^. However, reported heritability estimates for AGD score in Tasmanian population showed higher range with estimates of 0.10 to 0.48^7,8,15–17^, with lower heritability estimates obtained from the first infection and the higher estimates for the subsequent infections. Tasmanian research has shown that the resistance against first and later subsequent infections are different traits with poor genetic correlations (average ${r}_{g}$= 0.24)^16^. Selective breeding has been effective to increase the intervals between two consecutive baths/treatments which lead to overall reduction in number of baths/treatments and ultimately reduction in the expenses incurred on AGD^16^. However, addition of AGD resistance in breeding goal traits will reduce selection response for other traits, particularly when AGD show unfavourable genetic correlations to any other traits^18,19^. The use of marker assisted selection (MAS) and/or genomic selection (GS) using molecular markers that are directly or indirectly linked to variation in causal loci could provide potent tools to overcome these challenges which may increase both selection accuracy as well as selection intensity as this allow also for within family selection^20,21^. In addition, identification and subsequent fine-mapping of QTL regions should allow for the pinpointing of genes that underlie such traits. Significant association between genetic markers and quantitative traits of economic importance have been reported in Atlantic salmon^22–24^.

A few studies have been conducted on detection of QTL for resistance against AGD, where results have shown that the genetic architecture for AGD resistance trait is polygenic in nature^8,9^. Moreover, the results on transcriptomic profiles of Atlantic salmon in response to AGD infection seems to also explain polygenic nature of this trait with changes in expression of many genes in infected individuals^9,25^. The genes with functional properties in the immune system (e.g. interleukin-1 beta, a pro-inflammatory cytokine)^9,26,27^ as well as in cellular-adhesion (e.g. CCAAT/enhancer binding protein beta)^9,25^ were reported to be of importance for playing role in trait variation.

Availability and popularity of advanced GS by which breeding values of individuals are predicted using statistical methods has become a method of choice in recent era. The GS involves genotypic data for genome-wide distributed single nucleotide polymorphism (SNP) markers^28^ and provides opportunity to rank individuals within and across families. The feasibility of GS depends on the availability of a high-quality SNP genotyping platform and on extensive trait records collected in the reference populations. It has already been a widely used approach in many livestock and aquaculture species^29–33^ due to relative reduction in genotypic and sequencing costs which is primarily applied for the improvement of traits of economic and welfare importance (e.g. disease resistance).

The aim of the current study was to identify the genetic basis of host resistance to AGD by performing genome-wide association analysis (GWAS), and compare accuracies of genomic vs. pedigree-based predictions.

## Materials and Methods

### Resource population

The population used originated from Marine Harvest (MH) breeding nucleus in Ireland which was developed with a cross of parents mated in 1:2 male to female ratio. The starting population had 150 full sib families with 40 full-sibs per family. Families were communally reared from the eyed egg stage, and the tagging was performed at an average body weight of ~45 g. At tagging, fin clip samples were collected and preserved in 100% ethanol for further DNA extraction and genotyping (~50 K SNP chip). Pedigree was constructed using a panel of 65 SNP markers.

### Field test

A population of 6,100 fish at an average weight of 61 g were placed in a sea net-cage on 24. April 2014 at the South West farm of Marine Harvest Ireland, where AGD is a common problem. Fish population was allowed to develop AGD from the natural concentration of amoeba in sea. In June 2014, AGD was reported at the farm holding the fish and presence of *P. perurans* was confirmed by PCR. The monitoring for the development of AGD in the test cage was done by regular gill-scoring of a small number (~10–15) of fish per week. Gills were scored from 0 to 5 as described by Taylor *et. al*.^7^ with intensity of infection increasing with ascending order of score level. Major scoring for AGD phenotypes (first infection) was done on 29^th^ July 2014 when the estimated average gill-score in the sea net-cage passed score 2. Fish were haphazardly picked from the cage and uniquely gill scored by one of the three scorers (A, B and C). The sampling continued until the daylight was no longer appropriate for gill scoring which resulted in 3,663 gill-scored fish. The day after the fish were treated with fresh water to kill the amoeba, after which the fish were allowed to develop another round of a natural AGD infection (second infection). Scoring for second infection was also done by the same three scorers on 12^th^ of September 2014, which resulted in 3,511 gill-scored fish.

### DNA extraction and genotyping

From the 3,663 gill-scored fish during first infection, 1,190 fish (10 sibs per family) were randomly selected from 119 full-sib families for further processing. Genomic DNA was extracted from the fin clips using a commercial kit (DNeasy Blood & Tissue Kit, Qiagen), following the manufacturer’s instructions. Fish were genotyped using a 57 K axiom Affymetrix SNP Genotyping Array (NOFSAL2). After genotyping, we were left with a total of 1334 (193 Parents + 1141 Progeny) individuals from 119 full-sib families of 6–10 offspring per family and the rest either failed to genotype or filtered out during genotype calls due to the poor genotype quality.

### Filtering of SNPs

Genotypic data was filtered using the Plink software^34^, and SNPs were excluded with minor allele frequency (MAF) lower than 5%, missing rate higher than 15%, and Mendelian errors. Finally, approximately 54 K SNPs were retained for analyses.

### Statistical analysis

Analyses were performed for both first and second infection but the second infection did not show any genetic variation for this dataset of 1,141 phenotypes. Hence, following analyses were continued with gill-scores obtained in first infection only.

The applied model also included a fixed effect of scorer and an additional random effect common to full-sibs other than additive genetics. However, both effects were found to be non-significant (P > 0.05) and was therefore omitted from the model.

#### Genome wide association analysis (GWAS)

Genome wide association analysis was performed using the following linear mixed animal model implemented in GCTA program with “–mlma-loco” and “–reml” functions^35^.

$$y=\mu +X\alpha +Zu+e$$

where $y$ is a vector of n (n = 1,141) AGD scores, $\mu$ is an overall mean; $X$ is the incidence matrix for SNP containing marker genotypes coded as $0=AA,1=AB|BA,2=BB$, $\alpha$ is the allele substitution effect of each SNP, $Z$ is the incidence matrix of genotyped individuals, $u$ is the vector of genomic breeding values with $u\sim N(0,G{\sigma }_{u}^{2}),\,$where ${\sigma }_{u}^{2}\,$is the additive genetic variance, and $e$ is the vector of random residual effects with $e\sim N(0,I{\sigma }_{e}^{2})$. The G matrix is a genomic relationship matrix (GRM) which was computed according to VanRaden (2008)^36^ as $\frac{ZZ{\prime} }{2\ast {\sum }_{i=1}^{Nsnp}\,{p}_{i}(1-{p}_{i})};$ where ${p}_{i}\,$is the allele frequency of second allele and $Nsnp$ is the total number of SNP markers.

SNPs were considered genome wide significant when they exceeded the Bonferroni threshold^37^ for multiple testing (alpha = 0.05) of $0.05/tg$, where $tg$ =53,865 (total number of SNPs genome-wide) and graded as chromosome-wide significant when Bonferroni threshold for multiple testing surpassed (alpha = 0.05) $0.05/tc$, where $tc$ =1,796 (average number of SNPs per chromosome). Genome-wide significant threshold used in this study was considered to be $P\le 9.28\,\times 10{}^{-7}$ which is equivalent to $-lo{g}_{10}(P)=6.03$, while chromosome-wide significant threshold was opted to be $P\le 2.78\times {10}^{-5}$ which is equal to $-lo{g}_{10}(P)=4.55$

Quantile-quantile (q-q plot) plot with distribution of observed vs. expected p-values was checked, and the Inflation factor (lambda, λ) was calculated using following equation

$$lambda(\lambda )=\frac{median({\chi }^{2})}{0.456}$$

#### Estimation of SNP variances

Variances explained by the top significant SNP(s) were estimated using following two approaches (direct and indirect).

For the direct approach, variances explained by the top significant SNP(s) were estimated as $=2{p}_{i}{q}_{i}{\alpha }_{i}^{2}$ (Falconer and Mackay (1996)^38^). Therefore, the proportion of the of genetic (%$var{G}_{SN{P}_{i}}$) or phenotypic (%$var{P}_{SN{P}_{i}}$) variances captured by these markers equals $\frac{va{r}_{SN{P}_{i}}}{{\sigma }_{g}^{2}}\times 100$ and $\frac{va{r}_{SN{P}_{i}}}{{\sigma }_{p}^{2}}\times 100$, respectively. Where, ${p}_{i}$ and ${q}_{i}$ are allele frequencies for the major and the minor alleles respectively, whereas ${\sigma }_{g}^{2}$ and ${\sigma }_{p}^{2}$ are the genetic and phenotypic variances computed with the above animal model using genomic relationship matrix.

For the indirect approach, the proportion of the genetic or phenotypic variance explained by the genome-wide significant SNP(s) was estimated using the model: $y=\mu +GWS+Zu+e$. Where, $GWS$ are the genome wide significant SNP(s), the $G$ matrix used in this model was constructed with all other SNPs except genome-wide significant SNP ($GWS$). The variance (genetic or phenotypic) explained by the $GWS$ SNPs was expressed as a reduction in the total genetic or phenotypic variance.

#### Breeding value estimation

Pedigree as well as genomic breeding values (PEBVs vs. GEBVs) were computed using full (n=3,663) or reduced (n=1,141) datasets. The full dataset contained phenotypic records on all the recorded animals (n=3,663), while the reduced dataset (n=1,141) included phenotypic records on only the genotyped individuals which is a subset of the full data. Breeding values were estimated by applying the same model as described under the “GWAS” section of materials and methods, except that the marker effect ($$X\alpha $$) was excluded from the model and the genomic relationships ($$G)$$ was constructed using all SNPs that passed quality control. Breeding values for all scenarios were computed using ASreml v4.0^39^ program. Pedigree-based breeding values were computed by replacing the G matrix with the numerator relationship matrix (A). Pedigree breeding values were obtained with the dataset consisting of phenotypic records from only the genotyped (PBLUP_I) or from all the phenotyped (PBLUP_II) animals. Similarly, genomic breeding values were computed using records from only the genotyped animals (GBLUP) or a combined relationship matrix that uses all genotyped and phenotyped (ssGBLUP) animals. Whereas the G matrix was used for the GBLUP analysis, the realized relationship matrix (H) replaces G. The inverse of the H matrix (Legarra *et al*., 2009; Misztal *et al*., 2009) was constructed as follows:

$${H}^{-1}={A}^{-1}+[\begin{array}{cc}0 & 0\\ 0 & {(0.95G+0.05{A}_{22})}^{-1}-{A}_{22}^{-1}\end{array}]$$

where $G$ is as described above and ${A}_{22}\,$is the pedigree-based relationship matrix for genotyped animals. The variance components (${\sigma }_{u}^{2}=0.120$ and ${\sigma }_{e}^{2}=0.480)$ used for the genomic prediction analysis were computed from the full dataset^15^ and was fixed in all analysis.

#### Cross-validation and accuracy of prediction

Within family cross-validation scheme was used to assess the accuracy of the predicted breeding values. The phenotypes of four offspring per sire family were randomly masked as validation dataset (296 offspring out of 1,141) and the remaining animals were used as training dataset. This procedure was replicated 50 times and for each replicate, accuracy of predictions were computed as:

the correlation ($${r}_{corr})$$ of either the estimated pedigree (PEBV) or the genomic (GEBV) breeding value with the pre-corrected phenotype $${y}_{adj}$$ which was scaled by the square root of heritability as $${r}_{corr}=\frac{\rho (G[P]EBV,{y}_{adj})}{\surd {h}^{2}}$$; where $$\rho $$ = correlation coefficient, $$G[P]EBV$$ = breeding values estimated using genomic (GBLUP) or pedigree (PBLUP) information; $${h}^{2}$$ = $$0.20\pm 0.03$$ as reported by Lillehammer *et al*. 2019^15^.

## Results

### Descriptive statistics

**Figure 1:**
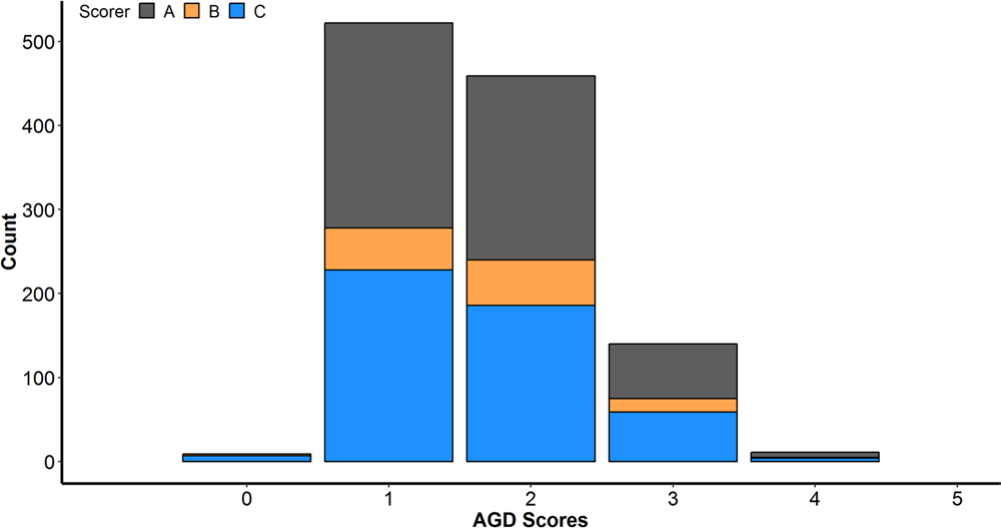
Distribution of AGD gill-scores and frequency of scoring by each scorer.

The 1,141 gill-scored fish had a mean gill-score of 1.67 and standard deviation of 0.73. The distribution of the gill-scores and number of individuals scored by each scorer is given in Fig. 1. The number of fish scored by each person was 123, 484, and 534 (B, C and A, respectively), and the gill-scores ranged from 0–4 with very low frequency of the extreme phenotypes (gill-scores 0 and 4).

### Genomics based variance components

Estimated variance components using narrated models with and without the genome-wide significant SNP are given in Table 1. Genomic heritability for resistance to AGD was 11.4%, but was reduced to 7.1%, or by 37.7%, when the genome-wide significant SNP (see Table 2) were accounted for in the model.

**Table 1:** Estimates of variance components and heritability with standard errors (in parenthesis) using the genomic relationship matrix. ${\sigma }_{g}^{2}$ = Genetic variance; ${\sigma }_{p}^{2}$ = Phenotypic variance; ${\sigma }_{e}^{2}$ = Residual variance; ${h}^{2}$ = Heritability; 1 = Model without any SNP used as fixed effect; 2 = Model with genome-wide significant SNP used as fixed effect.

Model	${{\boldsymbol{\sigma }}}_{{\bf{g}}}^{{\bf{2}}}$	${{\boldsymbol{\sigma }}}_{{\bf{e}}}^{{\bf{2}}}$	${{\boldsymbol{\sigma }}}_{{\bf{p}}}^{{\bf{2}}}$	Genomic *h*^2^
1	0.061 (0.021)	0.472 (0.025)	0.533 (0.023)	0.114 (0.037)
2	0.036 (0.018)	0.467(0.025)	0.503 (0.023)	0.071 (0.036)

**Table 2:** The top five significant SNPs detected in GWAS analysis ranked with respect to level of significance. Ssa = *Salmo salar* chromsomes; Pos(bp) = Physical position of SNP; A1 & A2 = Minor & major alleles, respectively; MAF = Minor allele frequency; α = Allele substitution effect; SE = Standard error; P = Significance value; $varP$ = Phenotypic variance explained; $varG\,$(%) = Proportion of genotypic variance explained; $varP$(%) = Proportion of phenotypic variance explained.

SNP-ID	Ssa	Pos(bp)	A1	A2	MAF	α	SE	P	${\boldsymbol{V}}{\boldsymbol{a}}{\boldsymbol{r}}{\boldsymbol{P}}$	${\boldsymbol{v}}{\boldsymbol{a}}{\boldsymbol{r}}{\boldsymbol{G}}\,$(%)	${\boldsymbol{v}}{\boldsymbol{a}}{\boldsymbol{r}}{\boldsymbol{P}}$ (%)
AX-88266207	02	1967633	A	B	0.165	−0.265	0.052	4.12E-07	0.019	32.6	3.63
AX-87970438	05	76572428	A	B	0.184	−0.228	0.050	5.73E-06	0.016	26.3	2.92
AX-88137791	02	2059898	B	A	0.184	−0.208	0.047	7.96E-06	0.013	22.0	2.45
AX-87975635	01	52913027	A	B	0.314	0.159	0.037	2.08E-05	0.011	18.4	2.05
AX-87017245	01	53002456	A	B	0.313	0.159	0.037	2.13E-05	0.011	18.3	2.04

### Genome wide association analysis and SNP variances

**Figure 2:**
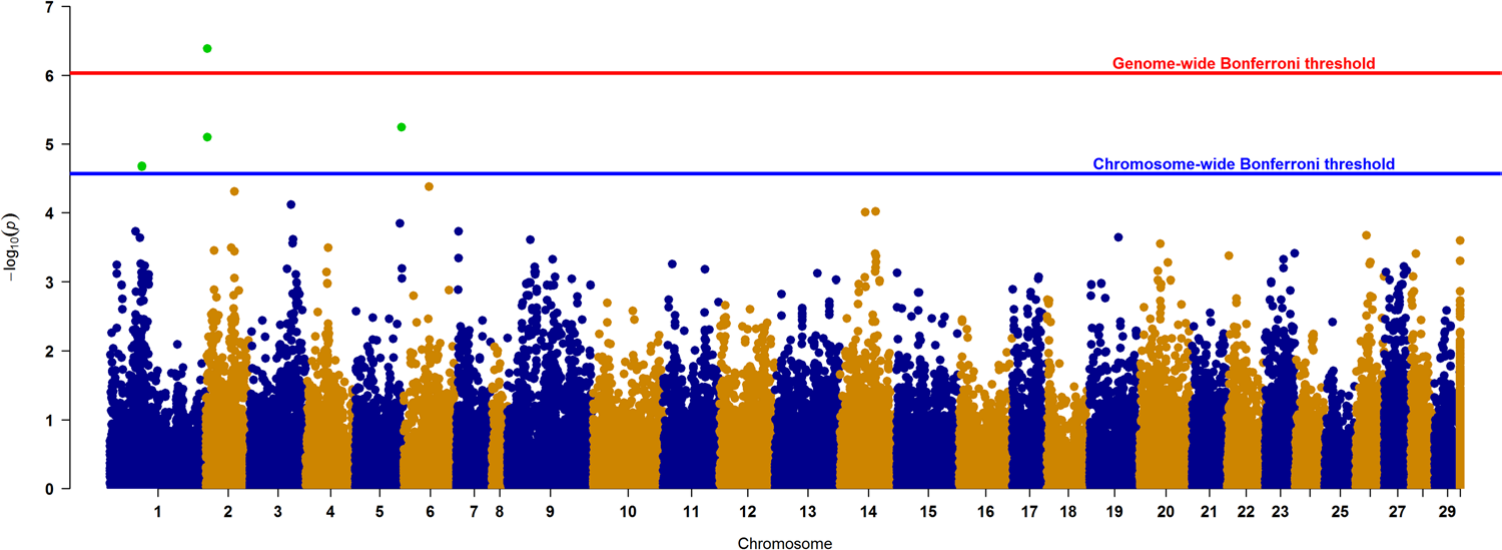
Manhattan plot of GWAS with p-values distributed across different chromosomes. Markers crossing genome and/or chromosome wide Bonferroni threshold are dotted in green color. Chromosome 30 represent markers belonging to unknown chromosome(s).

GWAS analysis using 53,865 SNPs on 1,141 recorded individuals resulted in a total of 5 SNPs which crossed genome or chromosome-wide significant level (Fig. 2). These five most significant SNPs are distributed across three different chromosomes 1, 2, and 5 (Ssa01, Ssa02 and Ssa05); with 2, 2 and 1 SNPs respectively (Table 2). The λ value (i.e. magnitude of the deviation inflation/deflation of p-values) for the analysis was recorded to be 1.094, and distribution of p-values are presented in a Q-Q plot (Supplementary Fig. S1.1).

 Phenotypic variance and the proportion of genetic and phenotypic variances explained by each of the five most significant SNPs are shown in Table 2. The proportion of the genetic variation (estimated with the direct approach) captured by each of these SNPs ranged from 18.3–32.6%. While the proportion of genetic variance explained by the genome-wide significant SNP estimated with the indirect method was 37.7%.

Potential genes with effect on AGD were searched around ±20 Kb region of the genome or chromosome-wide significant SNPs using salmon genome database^40^. The highest significant SNP of chromosome 2 was annotated as an intronic SNP located within the intron of “*tnxb.b”* gene and another gene “*c4”* is located upstream to this SNP (Table 3, Fig. S1.2). Both genes (*tnxb.b* and *c4*) are known to play a role in the immune system^41^.

**Table 3:** Summary for the functions of important candidate genes at QTL region. Genes are searched within approximately ± 20Kb region of the significant SNPs, and detected candidate genes might be playing role in variation for AGD phenotype.

Ssa	SNP	Annotation	Genes (~ ± 20 Kb)	Description	Ref
02	AX-88266207	Intronic	*tnxb.b*	This gene encodes a member of the tenascin family of extracellular matrix glycoproteins. This protein is thought to function in matrix maturation during wound healing.	^41^
			*slc45a4*	Solute carrier family 45, member 4, found to be functioning as sucrose transporter	^54^
			*c4*	It is central to the activation of both the classical and the lectin pathways of complement activation. The complement system is a part of the immune system that enhances (complements) the ability of antibodies and phagocytic cells to clear pathogens from an organism.	^45^
05	AX-87970438	Intronic	*slc44a4*	Solute carrier family 44 member 4 involved in the uptake of choline by cholinergic neurons.	^41^
			*ano10a*	The transmembrane protein encoded by this gene is a member of a family of calcium-activated chloride channels.	^41^
01	AX-87017245	3’UTR	*kcng3*	Potassium voltage-gated channel modifier subfamily G member 3. It is diverse in functionality includes epithelial electrolyte transport, heart rate etc.	^55^

### Accuracy of prediction

Pedigree and genomic-based prediction accuracies for amoebic gill disease are given in Table 4. Overall, genomic (GBLUP and ssGBLUP) based accuracies (${{\boldsymbol{r}}}_{{\boldsymbol{cor}}}$) were higher compared to using pedigree information (PBLUP_I and PBLUP_II). Accuracies from the model that used phenotypes (n=1,141) of only genotyped individuals (PBLUP_I and GBLUP) was much lower than using phenotype information (n = 3,663) from all individuals (PBLUP_II and ssGBLUP) that were recorded (Table 4). The increase in accuracy (${{\boldsymbol{r}}}_{{\boldsymbol{cor}}}$) when the number of phenotypic records tripled (n = 1141 vs n = 3,663) was about 34%. The blending of genomic and pedigree information led to about 12% increase in accuracy (ssGBLUP =0.47 vs PBLUP_II = 0.43).

**Table 4:** Genomic vs. pedigree based prediction accuracies for amoebic gill disease. PBLUP_I and PBLUP_II – Pedigree based breeding values using phenotypes from only genotyped or all phenotyped animals, respectively. GBLUP - Genomic breeding values from only genotype animals, and ssGBLUP - Genomic breeding values from all genotyped and phenotyped animals obtained with a combined relationship matrix (H).

Model	Number of fish			Accuracy
	Total	Training	validation	
PBLUP_I	1,141	845	296	0.32_(0.10)_
GBLUP	1,141	845	296	0.35_(0.09)_
PBLUP_II	3,663	3,367	296	0.43_(0.11)_
ssGBLUP	3,663	3,367	296	0.47_(0.12)_

### Ethical approval for the use of animals in this study

Although animals were used in this work, no direct experiments were performed on them. Permission was taken to use the site (InishFanard, on the south west coast of Ireland) for farming and production, and fish were moved to the site after obtaining the approval. Health status of fish was monitored regularly, and in-case of any outbreak fish were recorded and treated as part of routine practices. The studied population faced natural outbreak of AGD twice, and the recording of phenotype(s) is a routine procedure of breeding companies. The collection of tissue samples was carried out by highly skilled and experienced personnel from the breeding company. Tagging of fish, and the sampling the fin clips is not considered as an experimental intervention in the EU. Hence, no approval from the ethics committee was necessary according to local legislation.

## Discussion

Amoebic gill disease (AGD) is an increasing threat for the Atlantic salmon industry and understanding genetic basis of AGD and the application of advanced selection methods could lead to robust and sustainable salmon production. Current study aimed at detection of QTL(s) for AGD resistance as well as determine consistency in accuracy of genomic vs. pedigree based prediction methods.

The model applied in our statistical analyses did not include scorers as fixed effect because it was found to be non- significant (P > 0.05) for this dataset. However, scoring system for determining the severity of AGD is subjective, and it is therefore recommended to include scorer-effect in the model as it might have a significant effect as seen in some other experiments^15^. The observed genomic heritability of ~11.5% (for AGD score at first infection) obtained from our analysis was found to be lower than the previously reported estimates by Lillehammer *et al*.^15^ with 20% and 11% in field and challenge conditions respectively, and a field test based heritability of 14% (first infection) reported by Kube *et al*.^16^. The reported heritability estimates on AGD scores recorded at the third infection in challenge conditions fall within medium to higher level with estimates ranging from 0.24 to 0.48^7,8^. The difference in estimates could have been due to multiple reasons e.g. difference in adopted methodology for the estimations, population differences, infection types (first vs. subsequent later infections) used as phenotype which might activate different immune responses, e.g. first infection should activate innate while subsequent later infection should be mainly pursued by acquired immune system, and/or the total number of markers used in this study perhaps could not explain the total genetic variance.

The detected significant QTLs at chromosomes Ssa01, Ssa02 and Ssa05 for the AGD scores after first infectiondid not show concordance with the detected QTLs in previous studies of Robledo *et al*. and Boison *et al*.^8,9^. This disagreement could likely be due to the differences in infection conditions (challenge test in both studies vs natural field outbreak in current study), infection type or time of recording (first vs. subsequent infections), and/or differences in populations. The studied populations in both Robledo *et al*. and Boison *et al*.^8,9^ were challenge tested and had a trait in common where amoebic load was recorded using qPCR based ${{\boldsymbol{C}}}_{{\boldsymbol{t}}}$ values. The QTL results from Robledo *et al.* and Boison *et al.*^8,9^ also did not show concordance in any of the detected QTLs which further highlights the complexity of this trait, and perhaps indicates the cruciality of factors i.e. environment, time and type of recordings and genetic background of populations.

The top three most significant SNPs of GWAS analysis showed a favorable effect on the trait with negative α values which represent a reduction in AGD score (Table 2), while significant SNPs on Ssa01 had unfavorable effect on the trait (Figure S1.4, Table S1.1) with positive α values. Individual SNP specific genetic variances estimated using direct method (Table 2) cannot be added to find their cumulative effect because the top 3 SNPs on Ssa02 and Ssa05 are in high linkage disequilibrium (LD) with LD values ranging from 0.77–1.0, and the two SNPs on Ssa01 are also in complete LD of 1.0 (Supplementary Table S1.1 & Figure S1.4). Rather an average of the individual SNP genetic variances is likely to provide a better estimate which means that the top 3 SNPs and the significant SNPs on Ssa01 account for 26.9% and 18.3% of the genetic variance for resistance to AGD, respectively. However, as the indirect method (SNP as a fixed effect in the model) yielded a higher genetic variance estimate (~37%) which indicates that the individual SNPs are not completely linked as also indicated by the LD values less than unity. Application of indirect method with fixing the genome-wide significant SNP in the model appeared to cause insignificance of previously chromosome-wide significant SNPs of Ssa02 and Ssa05, and also shrinkage in p-values for all the other SNPs (Supplementary Figure S1.7). High shrinkage of p-values for “AX-87970438” and “AX-88137791” SNPs can be explained due to the existence of high co-linearity among the top 3 SNPs. Moreover, this high observed shrinkage in p-values of “AX-87970438” and “AX-88137791” SNPs also justifies the above described averaging function in-case of direct method instead of additive when estimating variances. The estimatedvariances explained by these significant (genome-wide and/or chromosome-wide) SNPs are likely to be inflated and could be due to the Beavis effect^42^. Large impact of these SNPs on genetic variation does not necessarily mean that the SNPs are causative mutations, but that these SNPs explain an important amount of the QTL variation, either directly or through LD with the causative mutations.

It is interesting to note that the significant SNPs on Ssa02 are located within ~92 Kb region (Figs. S1.2–1.4) and show high LD of 0.77, but the SNPs “AX-88137791” and “AX-87970438” located on chromosome Ssa02 and Ssa05, respectively showed complete LD of 1.0. The LD information among the significant SNPs in Figure S1.4 and Table S1.1 gives strong impression of co-segregation pattern for the SNPs on Ssa02 and Ssa05. We checked if SNPs were belonging to the homeologous regions of Ssa02 and Ssa05^40^, and interestingly they were positioned in homeologous block, 2p-5q_1 of both chromosomes (Ssa02 and Ssa05). However, when their positions were checked towards a recently developed linkage map (unpublished data), all three significant SNPs of Ssa02 and Ssa05 were found to be closely located on the same chromosome Ssa02 within a distance of 0.62 cM. The LD pattern and linkage map suggest that the top significant SNPs of Ssa02 and Ssa05 are located at the same chromosome which is discordant with the physical map and could be due to assembly error or complicated long-range linkage disequilibria explained by Koch *et al*.^43^. Manhattan plot for all chromosomes and the distribution of P-values for the SNPs on Ssa02 were replotted after correcting positions of significant markers (Figure S1.5 and S1.6) which provided relatively better shape of the QTL peak.

The region of ±20 Kb surrounding significant SNPs on Ssa02 include genes *tnxb.b* and *c4* which have been reported to be involved in the immune response (Table 3), and may play a role in AGD resistance. One of the SNPs on Ssa02 (the highest significant SNP of this study) is located within the intron of *tnxb.b* and this gene is reported to produce extracellular matrix glycoproteins which has anti-adhesive effects and is thought to function in matrix maturation during wound healing^44^. The *C4* gene is sandwiched between the two mutations located downstream (~38Kb distant) of the highest and upstream (~71.5 Kb distant) to the second highest SNPs of Ssa02. Gene *C4* is known to be central to the activation of both the classical and the lectin pathways of complement system that enhances the ability of antibodies and phagocytic cells to clear pathogens from an organism^45^. The functional properties of these candidate genes (*tnxb.b & C4*) with their role in immune system and in cellular-adhesion agrees with transcriptomics results of previous studies where immune and cellular-adhesion functionality genes were detected to be differentially expressed^9,25^. Available annotated information on both the genes (*tnxb.b & C4*) signifies the impact with strong signal that the QTL of Ssa02 could directly and/or indirectly be linked with variation in expression level and/or function of these gene(s), which ultimately cause variation in AGD score. However, further studies on QTL validations as well as advanced assays like *in situ* hybridization technique to detect localization of gene expression, differential expression or sequencing of selected genes in susceptible and resistance fish might lead to a better understanding of the biological mechanism/pathways in response to this pathogen.

Overall, we observed 9.3% increase in accuracy with genomic information depending on the method used to calculate the accuracies. Similar trend of higher accuracies using genomic vs. pedigree-based information has been reported in Atlantic salmon^46,47^ for parasite and pathogen resistance traits, as well as for production traits in livestock species^48–50^. The advantage of GBLUP over PBLUP is because realized genomic-based relatedness between animals deviate from pedigree-based relationship coefficients.

Our results showed that single-step methodology (ssGBLUP), which take advantage of pedigree, phenotypic and genomic information simultaneously, gave higher prediction accuracies compared to PBLUP and/or GBLUP, which used only pedigree or genomic information. Similar results obtained with ssGBLUP were reported in salmonids^51,52^ and cattle^53^.

## Conclusion

A SNP array based genotyping of a population of Atlantic salmon which was field recorded for resistance to AGD revealed one genome-wide significant and the two suggestive QTLs distributed over chromosome 1, 2 and 5. Three candidate genes; *c4*, *tnxb*, and *slc44a4* were found within nearly 20 Kb flanking region of the detected loci on chromosome 2. The *tnxb and c4* genes are known to be a part of innate immune system, which may be involved in resistance to AGD. Genomic prediction using SNP based genotypic data  improved prediction accracy with 9–17% over the pedigree-based predictions which highlights both the potential and importance of genomic selection in commercial breeding programs.

## Supplementary information

Supplementary file 1.

## Data Availability

Most of the data supporting these findings are contained within the manuscript. Specific queries regarding data can be made available upon request through corresponding author.
