# Supplementary material for: Genome-wide association mapping and accuracy of predictions for amoebic gill disease in Atlantic salmon (Salmo salar)
Source: Sci Rep. 2020 Apr 15;10:6435. doi: 10.1038/s41598-020-63423-8 (PMC7160127; doi:10.1038/s41598-020-63423-8)

**Genome-wide association mapping and accuracy of predictions for amoebic gill disease in Atlantic salmon (*Salmo salar*)**

*Muhammad L Aslam* ***^*1^****,* *Solomon A Boison* ***^1,2^****,* *Marie Lillehammer* ***^1^****, Ashie Norris* ***^2^****, Bjarne Gjerde* ***^1^***

***^1^*** *Department of Breeding and Genetics, Nofima AS, P.O. Box 210, N-1431 Ås, Norway*

***^2^*** *Marine Harvest Ireland ASA (old name) with new name Mowi Genetics AS, 5035 Bergen*

**Figure S1.1:** Q-Q plot for GWAS analysis.


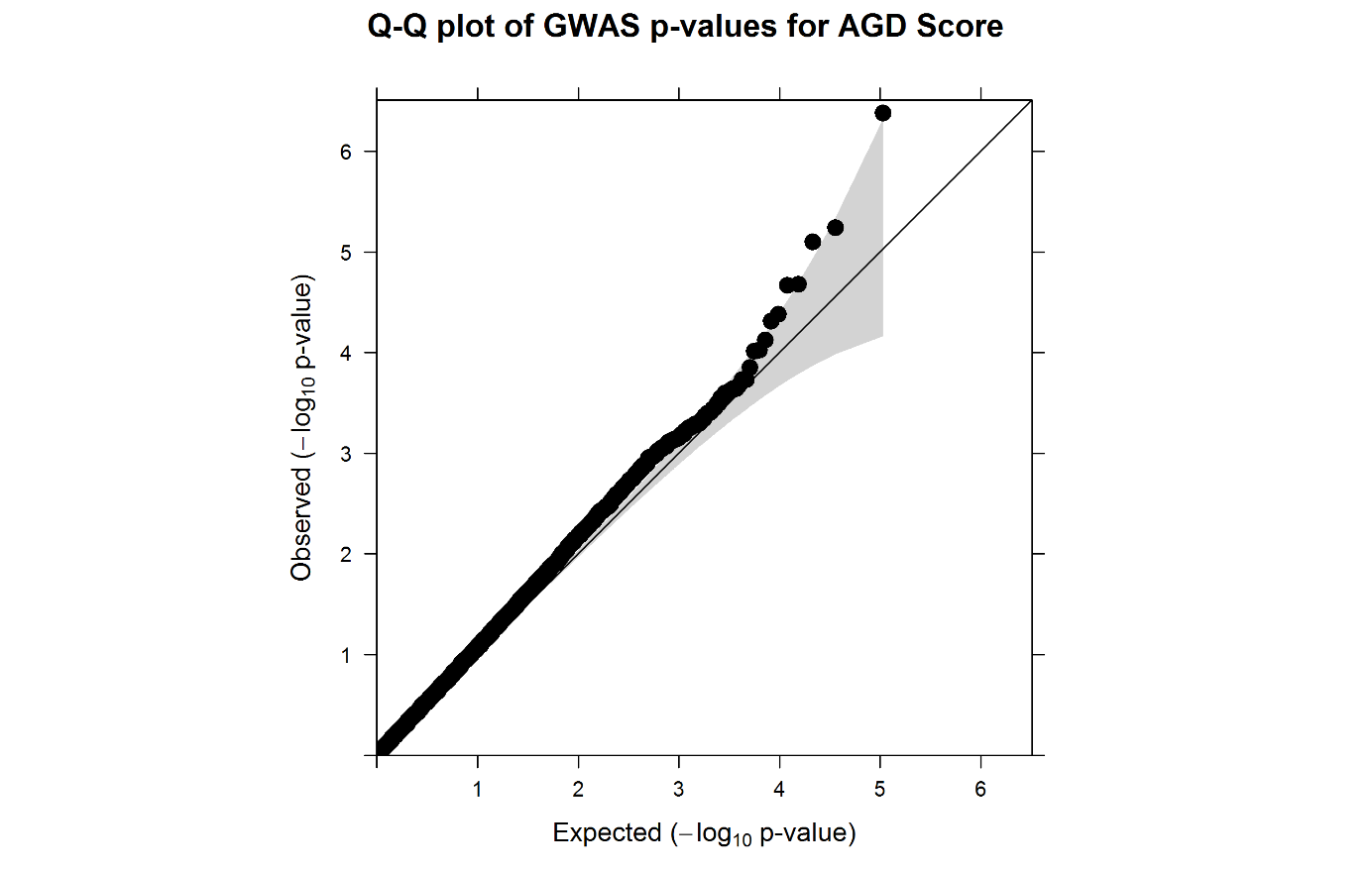

$$\lambda=1.094(se=7.8e^{05})$$

**
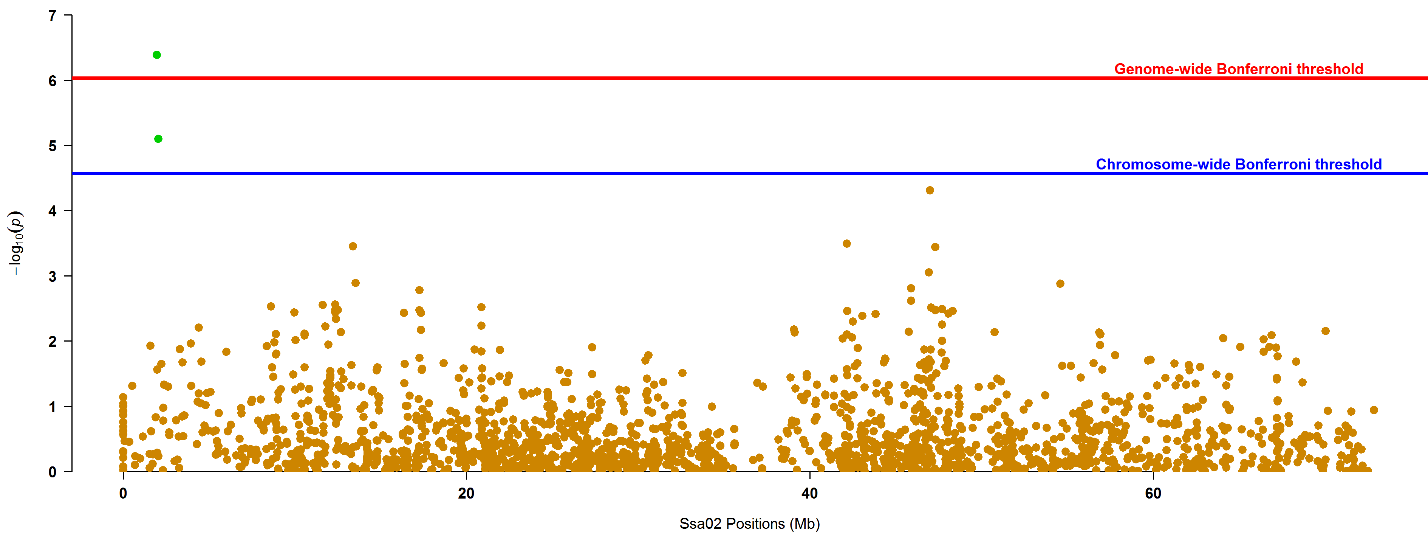
Figure S1.2:** P-values and position of top 2 significant SNPs of chromsome2.

**Figure S1.3:** Genes around the highest significant SNP of chromsome2.

**Figure S1.4:** Heat map of Linkage disequilibrium values for the top 5 significant markers in GWAS analysis.

**Table S1.1:** Linkage disequilibrium values of top 5 significant SNPs of GWAS analysis.

| **SNP-Ids** | **AX-87975635** | **AX-87017245** | **AX-88266207** | **AX-88137791** | **AX-87970438** |
| --- | --- | --- | --- | --- | --- |
| **AX-87975635** | 1,00 | 0,99 | 0,00 | 0,00 | 0,00 |
| **AX-87017245** |  | 1,00 | 0,00 | 0,00 | 0,00 |
| **AX-88266207** |  |  | 1,00 | 0,77 | 0,77 |
| **AX-88137791** |  |  |  | 1,00 | 1,00 |
| **AX-87970438** |  |  |  |  | 1,00 |

**Figure S1.5:** Manhattan plot after correcting positions using linkage and LD information


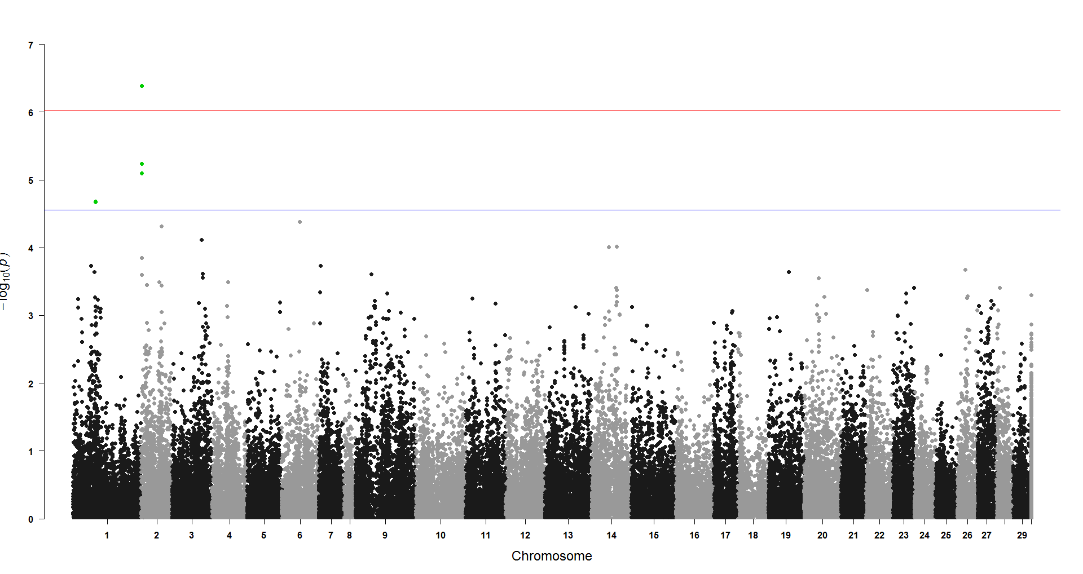


**
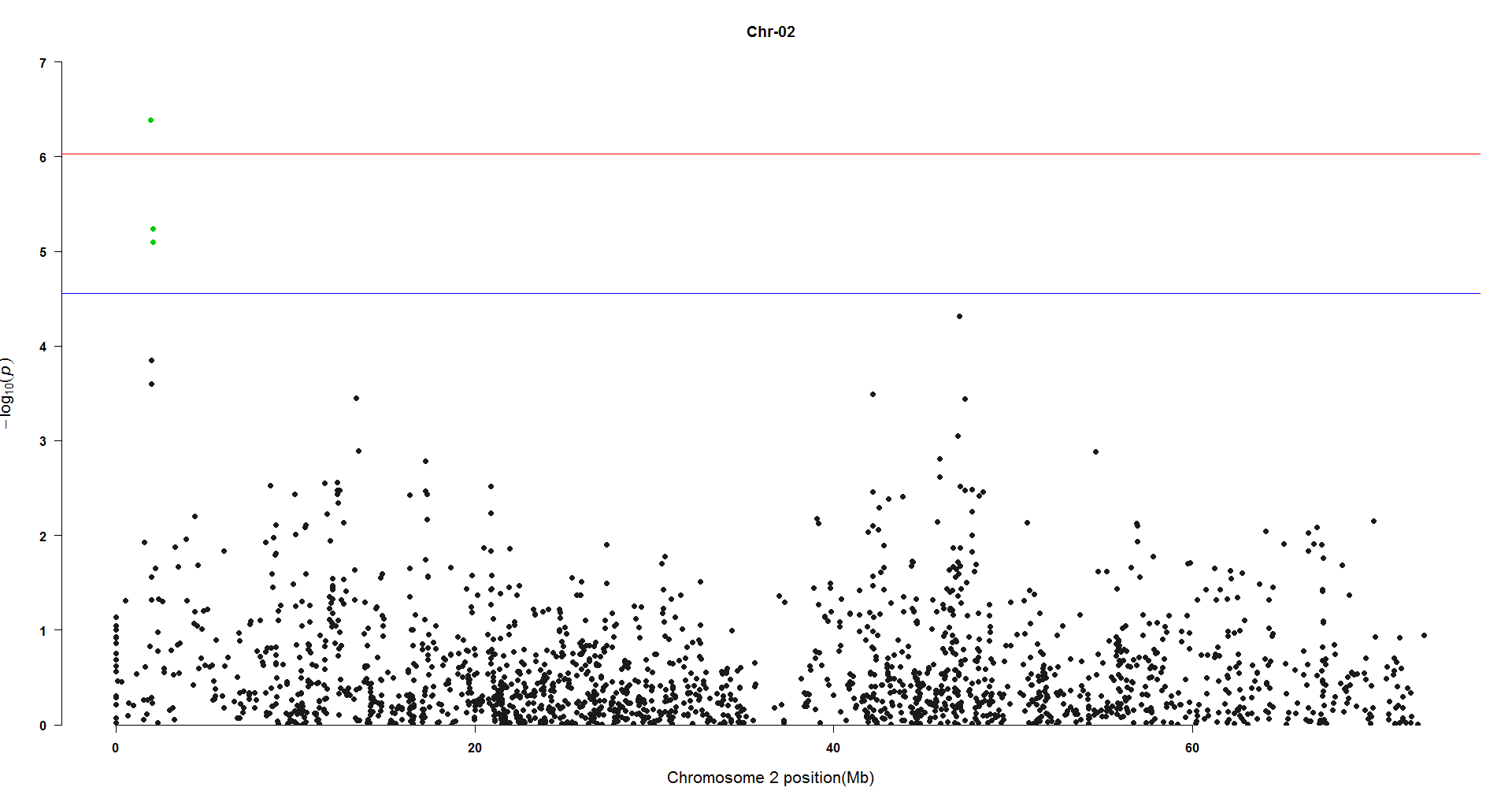
Figure S1.6:** Distribution of P-values at chromosome 2 (Ssa02) after correcting positions.

**Figure S1.7:** Manhattan plot showing shrinkage of p-values using model with the addition of top significant SNP used as fixed effect.


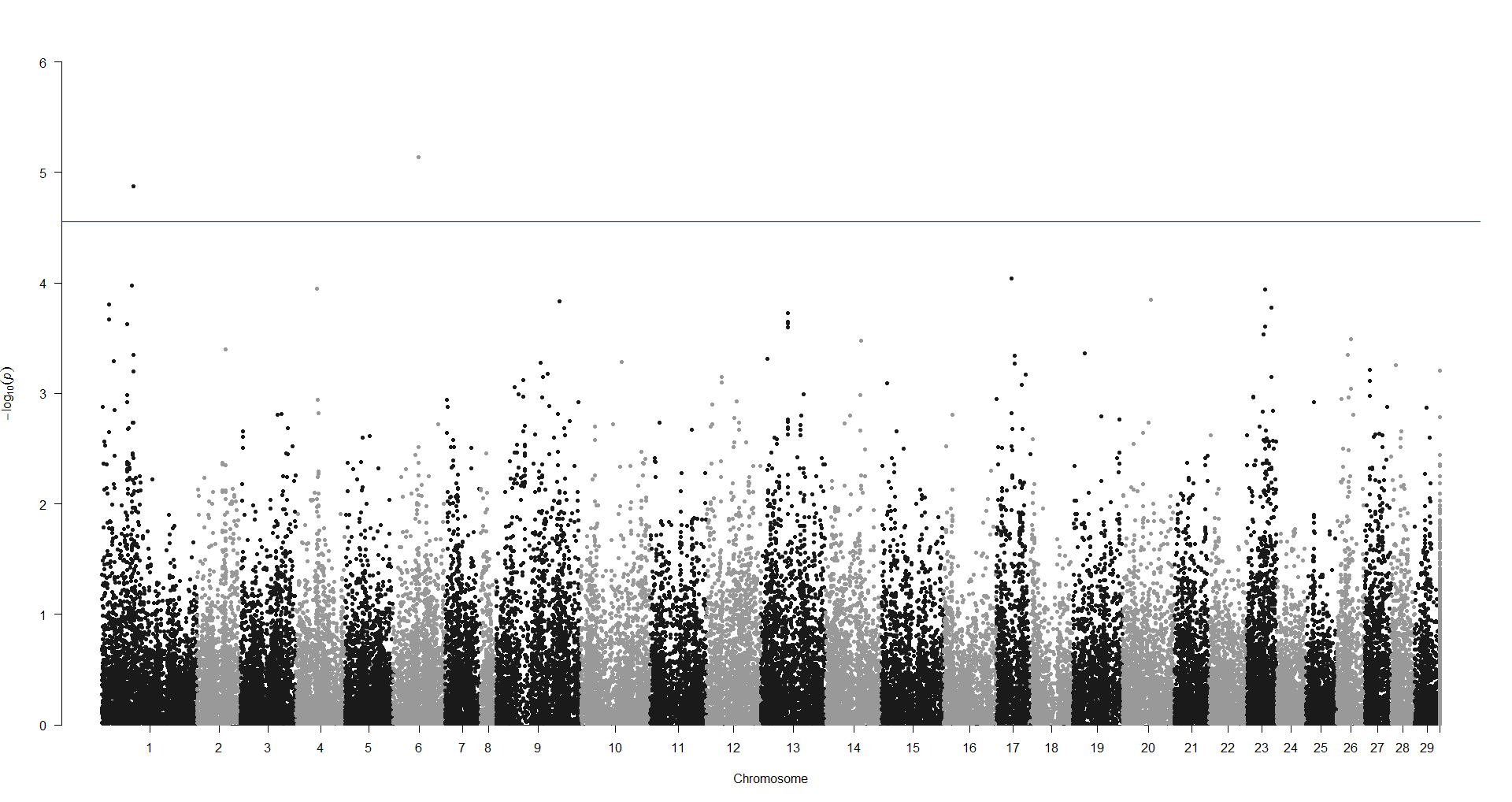

Supplement: Supplementary file 1 [file 41598_2020_63423_MOESM1_ESM.docx]
